# *N*,*N*′-Bis(2-quinolylcarbon­yl)hydrazine

**DOI:** 10.1107/S1600536809037842

**Published:** 2009-09-26

**Authors:** Hui-Min Cheng, Lang Chen, Wei Lu, Xuan Shen, Dun-Ru Zhu

**Affiliations:** a College of Chemistry and Chemical Engineering, State Key Laboratory of Materials-oriented Chemical Engineering, Nanjing University of Technology, Nanjing 210009, People’s Republic of China

## Abstract

The title compound, C_20_H_14_N_4_O_2_, crystallizes in the ortho­rhom­bic system with a crystallographic twofold axis through the N—N bond. The mol­ecule is non-planar and the dihedral angle between two amide groups is 74.9 (2)°. An intra­molecular N—H⋯N hydrogen bond is present. In the crystal, the mol­ecules are packed in chains running along the *c* axis through inter­molecular N—H⋯O hydrogen bonds. These chains are further stabilized by inter­molecular C—H⋯O hydrogen bonds and C—H⋯π inter­actions leading to the formation of a three-dimensional network.

## Related literature

For general background to the chemistry of *N*,*N*′-diacyl­hydrazines, see: Zhao & Bruke (1997 ▶); Knödler *et al.* (2004 ▶); Bernhardt *et al.* (2005 ▶). For the syntheses and structures of related compounds, see: Jasinskas *et al.* (1975 ▶); Shao *et al.* (1999 ▶); Xu *et al.* (2006 ▶); Zheng *et al.* (2007 ▶); Shanmuga Sundara Raj *et al.* (2000 ▶). For the synthesis of the title compound, see: Xie *et al.* (2009 ▶). For hydrogen-bond motifs, see: Bernstein *et al.* (1995 ▶);
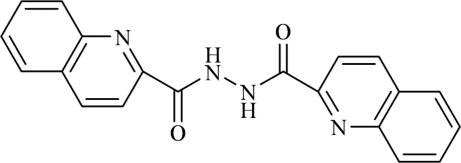


## Experimental

### 

#### Crystal data

C_20_H_14_N_4_O_2_
*M* *_r_* = 342.35
Orthorhombic, 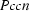

*a* = 11.649 (4) Å
*b* = 17.023 (6) Å
*c* = 8.349 (3) Å
*V* = 1655.6 (10) Å^3^
*Z* = 4
Mo *K*α radiation
μ = 0.09 mm^−1^
*T* = 296 K
0.26 × 0.12 × 0.08 mm

#### Data collection

Bruker APEXII CCD diffractometer
Absorption correction: multi-scan (*SADABS*; Bruker, 2005 ▶) *T*_min_ = 0.976, *T*_max_ = 0.993
10346 measured reflections
1629 independent reflections
816 reflections with *I* > 2σ(*I*)
*R*_int_ = 0.082

#### Refinement

*R*[*F* ^2^ > 2σ(*F* ^2^)] = 0.046
*wR*(*F* ^2^) = 0.115
*S* = 0.95
1629 reflections
119 parameters
H-atom parameters constrained
Δρ_max_ = 0.17 e Å^−3^
Δρ_min_ = −0.19 e Å^−3^

### 

Data collection: *APEX2* (Bruker, 2005 ▶); cell refinement: *SAINT* (Bruker, 2005 ▶); data reduction: *SAINT*; program(s) used to solve structure: *SHELXS97* (Sheldrick, 2008 ▶); program(s) used to refine structure: *SHELXL97* (Sheldrick, 2008 ▶); molecular graphics: *SHELXTL* (Sheldrick, 2008 ▶); software used to prepare material for publication: *SHELXTL*.

## Supplementary Material

Crystal structure: contains datablocks I, global. DOI: 10.1107/S1600536809037842/rz2361sup1.cif

Structure factors: contains datablocks I. DOI: 10.1107/S1600536809037842/rz2361Isup2.hkl

Additional supplementary materials:  crystallographic information; 3D view; checkCIF report

## Figures and Tables

**Table 1:** Hydrogen-bond geometry (Å, °)

*D*—H⋯*A*	*D*—H	H⋯*A*	*D*⋯*A*	*D*—H⋯*A*
N2—H2*A*⋯N1	0.86	2.31	2.689 (2)	107
N2—H2*A*⋯O1^ii^	0.86	2.35	2.978 (3)	130
C5—H5*A*⋯O1^iii^	0.93	2.45	3.177 (3)	135
C8—H8*A*⋯*Cg*1^iv^	0.93	2.64	3.449	146

Symmetry codes: (ii) 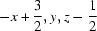; (iii) 
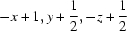
; (iv) 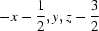. *Cg*1 is the centroid of the N1/C1–C4/C9 ring.

**Fig. 1.**
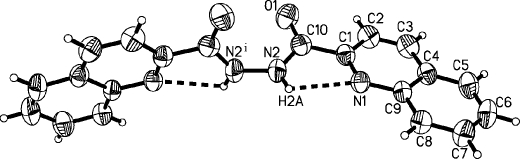
The structure of the title compound showing 50% probability displacement ellipsoids and atom-numbering scheme [symmetry code: (i) 3/2 - x, 1/2 - y, z].

**Fig. 2.**
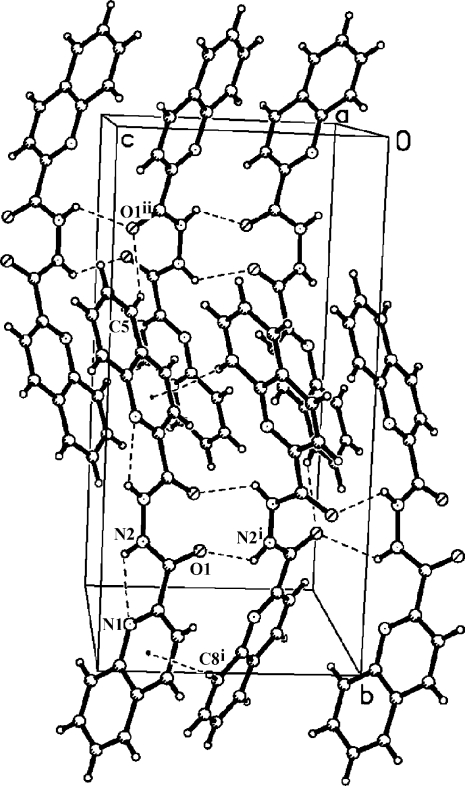
The three-dimensional network formed via hydrogen bonds (dashed lines) and C—H···π interactions [symmetry code: (i) 1 - x, 1/2 + y, 1/2 - z].

## supplementary crystallographic information

### Comment

(Un)symmetrical *N*,*N*'-diacylhydrazines are of interest because they are the basic structural components in heterocyclic chemistry and may be used as bridging ligands in coordination chemistry (Zhao & Bruke, 1997; Knödler *et al.*, 2004; Bernhardt *et al.*, 2005). We have reported the structure of *N*,*N*'-bis(2-picolinoyl)hydrazine (Shao *et al.*, 1999). As a continuation of our investigations of the structure of *N*,*N*'-diacylhydrazines and their derivatives, herein, we report the crystal structure of the title compound. It was first prepared by aroylation of 2-quinolylcarbonylhydrazine with 2-quinolinecarbonyl chloride in dry pyridine (Jasinskas *et al.*, 1975).

The X-ray analysis of the title compound (Fig. 1) indicates that the molecule is non-planar. The dihedral angle between the quinolyl ring and  the amide group is 15.3 (2)° and that between the amide groups is 74.9 (2)°. Similarly to *N*,*N*'-bis(2-picolinoyl)hydrazine, the asymmetric unit contains half the molecule and the other half is related by a crystallographic twofold axis passing through the N2—N2^i^ bond [symmetry code: (i) 3/2 - *x*, 1/2 - *y*, *z*]. The bond lengths and angles (Table 1) in the structure are in the normal ranges (Xu *et al.*, 2006; Zheng *et al.*, 2007). The C10—N2—N2^i^—C10^i^ torsion angle is -87.7 (2)°. The two carbonyl groups and the H atoms of the N—N bond are in a *trans* orientation with respect to each other. This conformation is due mainly to the intramolecular N—H···N hydrogen bonds.

In the crystal (Fig. 2), each molecule is connected to another by a pair of intermolecular N—H···O hydrogen bonds (Table 2) between the amide H atoms and the O atoms of neighbouring carbonyl groups to form a ten-membered ring with the graph-set motif *C*4*R*^2^_2_(10) (Bernstein *et al.*, 1995). The same feature is also found in *N*,*N*'-bis(2-picolinoyl)hydrazine and 1,2-dibenzoylhydrazine (Shanmuga Sundara Raj *et al.*, 2000). Due to presence of these intermolecular N—H···O hydrogen bonds, the molecules are packed into chains running along the *c* axis. These chains are further stabilized by intermolecular C—H···O hydrogen bonds and C—H···π interactions (Table 2) leading to the formation of a three-dimensional network.

### Experimental

The title compound was obtained unexpectedly in the synthesis of 3-methyl-4-(*p*-methylphenyl)-5-(2-quinolyl)-1,2,4-triazole by the reaction of *N*-formyl-*N*'-(2-quinolylcarbonyl)hydrazine (1 mmol) with 4,4'-dimethylphenylphosphazoanilide (1 mmol) in *N*,*N*-dimethylaniline (20 ml) at 463–473 K (Xie *et al.*, 2009). It also can be prepared by literature method (Jasinskas *et al.*, 1975). Diffraction quality crystals were obtained by recrystallization from ethanol (yield 31%).

### Refinement

All H atoms were located in a difference Fourier map and allowed to ride on their parent atoms at distances of 0.96Å (aromatic), 0.93Å (pyridyl), and with *U*_iso_(H) values of 1.2 or 1.5 times of *U*_eq_(C).

### Crystal data

**Table:**

C_20_H_14_N_4_O_2_	*F*(000) = 712
*M**_r_* = 342.35	*D*_x_ = 1.374 Mg m^−^^3^
Orthorhombic, *P**c**c**n*	Mo *K*α radiation, λ = 0.71073 Å
Hall symbol: -P 2ab 2ac	Cell parameters from 28 reflections
*a* = 11.649 (4) Å	θ = 2.1–26.6°
*b* = 17.023 (6) Å	µ = 0.09 mm^−^^1^
*c* = 8.349 (3) Å	*T* = 296 K
*V* = 1655.6 (10) Å^3^	Block, colourless
*Z* = 4	0.26 × 0.12 × 0.08 mm

### Data collection

**Table:**

Bruker APEXII CCD diffractometer	1629 independent reflections
Radiation source: fine-focus sealed tube	816 reflections with *I* > 2σ(*I*)
graphite	*R*_int_ = 0.082
ω scans	θ_max_ = 26.0°, θ_min_ = 2.1°
Absorption correction: multi-scan (*SADABS*; Bruker, 2005)	*h* = −14→14
*T*_min_ = 0.976, *T*_max_ = 0.993	*k* = −21→18
10346 measured reflections	*l* = −9→10

### Refinement

**Table:**

Refinement on *F*^2^	Secondary atom site location: difference Fourier map
Least-squares matrix: full	Hydrogen site location: inferred from neighbouring sites
*R*[*F*^2^ > 2σ(*F*^2^)] = 0.046	H-atom parameters constrained
*wR*(*F*^2^) = 0.115	*w* = 1/[σ^2^(*F*_o_^2^) + (0.0525*P*)^2^] where *P* = (*F*_o_^2^ + 2*F*_c_^2^)/3
*S* = 0.95	(Δ/σ)_max_ < 0.001
1629 reflections	Δρ_max_ = 0.17 e Å^−^^3^
119 parameters	Δρ_min_ = −0.19 e Å^−^^3^
0 restraints	Extinction correction: *SHELXL97* (Sheldrick, 2008), Fc^*^=kFc[1+0.001xFc^2^λ^3^/sin(2θ)]^-1/4^
Primary atom site location: structure-invariant direct methods	Extinction coefficient: 0.011 (2)

### Special details

**Table:**

Geometry. All e.s.d.'s (except the e.s.d. in the dihedral angle between two l.s. planes) are estimated using the full covariance matrix. The cell e.s.d.'s are taken into account individually in the estimation of e.s.d.'s in distances, angles and torsion angles; correlations between e.s.d.'s in cell parameters are only used when they are defined by crystal symmetry. An approximate (isotropic) treatment of cell e.s.d.'s is used for estimating e.s.d.'s involving l.s. planes.
Refinement. Refinement of *F*^2^ against ALL reflections. The weighted *R*-factor *wR* and goodness of fit *S* are based on *F*^2^, conventional *R*-factors *R* are based on *F*, with *F* set to zero for negative *F*^2^. The threshold expression of *F*^2^ > σ(*F*^2^) is used only for calculating *R*-factors(gt) *etc*. and is not relevant to the choice of reflections for refinement. *R*-factors based on *F*^2^ are statistically about twice as large as those based on *F*, and *R*- factors based on ALL data will be even larger.

### Fractional atomic coordinates and isotropic or equivalent isotropic displacement parameters (Å^2^)

**Table:**

	*x*	*y*	*z*	*U*_iso_*/*U*_eq_	
O1	0.61559 (14)	0.28662 (9)	0.3812 (2)	0.0651 (5)	
N1	0.69622 (14)	0.44375 (10)	0.1318 (2)	0.0424 (5)	
N2	0.73777 (18)	0.28973 (9)	0.1733 (2)	0.0605 (6)	
H2A	0.7745	0.3197	0.1080	0.073*	
C1	0.62868 (17)	0.40522 (13)	0.2314 (3)	0.0432 (6)	
C2	0.52811 (18)	0.43630 (14)	0.2994 (3)	0.0520 (7)	
H2B	0.4841	0.4063	0.3694	0.062*	
C3	0.49611 (18)	0.51056 (15)	0.2615 (3)	0.0549 (7)	
H3A	0.4290	0.5317	0.3037	0.066*	
C4	0.56521 (18)	0.55552 (12)	0.1576 (3)	0.0450 (6)	
C5	0.5394 (2)	0.63312 (13)	0.1127 (3)	0.0599 (7)	
H5A	0.4739	0.6573	0.1530	0.072*	
C6	0.6091 (2)	0.67300 (14)	0.0114 (3)	0.0663 (8)	
H6A	0.5908	0.7243	−0.0172	0.080*	
C7	0.7083 (2)	0.63781 (14)	−0.0508 (3)	0.0615 (7)	
H7A	0.7552	0.6658	−0.1205	0.074*	
C8	0.73620 (19)	0.56297 (13)	−0.0097 (3)	0.0498 (6)	
H8A	0.8025	0.5401	−0.0509	0.060*	
C9	0.66544 (17)	0.51985 (12)	0.0947 (3)	0.0401 (6)	
C10	0.65956 (19)	0.32223 (14)	0.2702 (3)	0.0471 (6)	

### Atomic displacement parameters (Å^2^)

**Table:**

	*U*^11^	*U*^22^	*U*^33^	*U*^12^	*U*^13^	*U*^23^
O1	0.0692 (11)	0.0517 (10)	0.0743 (13)	−0.0141 (9)	0.0059 (10)	0.0116 (10)
N1	0.0396 (11)	0.0382 (11)	0.0495 (12)	−0.0009 (8)	−0.0004 (9)	−0.0006 (9)
N2	0.0861 (15)	0.0349 (10)	0.0605 (14)	0.0080 (12)	0.0094 (12)	0.0031 (10)
C1	0.0407 (13)	0.0388 (13)	0.0501 (15)	−0.0023 (11)	−0.0059 (11)	−0.0051 (11)
C2	0.0404 (14)	0.0547 (17)	0.0610 (17)	−0.0066 (12)	0.0063 (12)	−0.0029 (13)
C3	0.0387 (13)	0.0605 (18)	0.0657 (17)	0.0031 (12)	0.0043 (12)	−0.0063 (14)
C4	0.0379 (13)	0.0428 (14)	0.0543 (16)	0.0046 (10)	−0.0049 (11)	−0.0065 (12)
C5	0.0508 (15)	0.0484 (16)	0.080 (2)	0.0138 (13)	−0.0026 (14)	−0.0058 (15)
C6	0.0691 (18)	0.0403 (15)	0.089 (2)	0.0079 (13)	−0.0075 (16)	0.0060 (15)
C7	0.0599 (16)	0.0489 (16)	0.076 (2)	−0.0021 (13)	0.0032 (14)	0.0091 (14)
C8	0.0459 (14)	0.0458 (15)	0.0577 (16)	0.0004 (11)	0.0014 (11)	0.0027 (12)
C9	0.0362 (12)	0.0358 (12)	0.0483 (14)	0.0012 (10)	−0.0064 (11)	−0.0033 (11)
C10	0.0489 (14)	0.0433 (15)	0.0491 (15)	−0.0100 (12)	−0.0030 (12)	−0.0013 (12)

### Geometric parameters (Å, °)

**Table:**

O1—C10	1.220 (3)	C3—H3A	0.9300
N1—C1	1.319 (3)	C4—C5	1.406 (3)
N1—C9	1.379 (2)	C4—C9	1.417 (3)
N2—C10	1.338 (3)	C5—C6	1.354 (3)
N2—N2^i^	1.382 (3)	C5—H5A	0.9300
N2—H2A	0.8600	C6—C7	1.401 (3)
C1—C2	1.405 (3)	C6—H6A	0.9300
C1—C10	1.493 (3)	C7—C8	1.359 (3)
C2—C3	1.355 (3)	C7—H7A	0.9300
C2—H2B	0.9300	C8—C9	1.406 (3)
C3—C4	1.409 (3)	C8—H8A	0.9300
			
C1—N1—C9	116.98 (18)	C6—C5—H5A	119.7
C10—N2—N2^i^	123.0 (2)	C4—C5—H5A	119.7
C10—N2—H2A	118.5	C5—C6—C7	120.7 (2)
N2^i^—N2—H2A	118.5	C5—C6—H6A	119.6
N1—C1—C2	124.4 (2)	C7—C6—H6A	119.6
N1—C1—C10	117.6 (2)	C8—C7—C6	120.3 (2)
C2—C1—C10	118.0 (2)	C8—C7—H7A	119.8
C3—C2—C1	119.1 (2)	C6—C7—H7A	119.8
C3—C2—H2B	120.5	C7—C8—C9	120.3 (2)
C1—C2—H2B	120.5	C7—C8—H8A	119.8
C2—C3—C4	119.5 (2)	C9—C8—H8A	119.8
C2—C3—H3A	120.2	N1—C9—C8	118.5 (2)
C4—C3—H3A	120.2	N1—C9—C4	122.2 (2)
C5—C4—C3	123.5 (2)	C8—C9—C4	119.3 (2)
C5—C4—C9	118.7 (2)	O1—C10—N2	122.7 (2)
C3—C4—C9	117.8 (2)	O1—C10—C1	122.3 (2)
C6—C5—C4	120.6 (2)	N2—C10—C1	115.1 (2)

Symmetry codes: (i) −*x*+3/2, −*y*+1/2, *z*.

### Hydrogen-bond geometry (Å, °)

**Table:**

*D*—H···*A*	*D*—H	H···*A*	*D*···*A*	*D*—H···*A*
N2—H2A···N1	0.86	2.31	2.689 (2)	107
N2—H2A···O1^ii^	0.86	2.35	2.978 (3)	130
C5—H5A···O1^iii^	0.93	2.45	3.177 (3)	135
C8—H8A···Cg1^iv^	0.93	2.64	3.449	146

Symmetry codes: (ii) −*x*+3/2, *y*, *z*−1/2; (iii) −*x*+1, *y*+1/2, −*z*+1/2; (iv) −*x*−1/2, *y*, *z*−3/2.
